# Cryo-EM Determination of Eravacycline-Bound Structures of the Ribosome and the Multidrug Efflux Pump AdeJ of Acinetobacter baumannii

**DOI:** 10.1128/mBio.01031-21

**Published:** 2021-05-28

**Authors:** Zhemin Zhang, Christopher E. Morgan, Robert A. Bonomo, Edward W. Yu

**Affiliations:** a Department of Pharmacology, Case Western Reserve University School of Medicine, Cleveland, Ohio, USA; b Louis Stokes Cleveland Department of Veterans Affairs Medical Center, Cleveland, Ohio, USA

**Keywords:** *Acinetobacter baumannii*, AdeJ, ribosome, eravacycline, multidrug efflux pump, multidrug resistance

## Abstract

Antibiotic-resistant strains of the Gram-negative pathogen Acinetobacter baumannii have emerged as a significant global health threat. One successful therapeutic option to treat bacterial infections has been to target the bacterial ribosome. However, in many cases, multidrug efflux pumps within the bacterium recognize and extrude these clinically important antibiotics designed to inhibit the protein synthesis function of the bacterial ribosome. Thus, multidrug efflux within A. baumannii and other highly drug-resistant strains is a major cause of failure of drug-based treatments of infectious diseases. We here report the first structures of the Acinetobacter drug efflux (Ade)J pump in the presence of the antibiotic eravacycline, using single-particle cryo-electron microscopy (cryo-EM). We also describe cryo-EM structures of the eravacycline-bound forms of the A. baumannii ribosome, including the 70S, 50S, and 30S forms. Our data indicate that the AdeJ pump primarily uses hydrophobic interactions to bind eravacycline, while the 70S ribosome utilizes electrostatic interactions to bind this drug. Our work here highlights how an antibiotic can bind multiple bacterial targets through different mechanisms and potentially enables drug optimization by taking advantage of these different modes of ligand binding.

## INTRODUCTION

The bacterial ribosome represents a major target for antimicrobial agents, as it is vital for protein synthesis and is one of the largest molecular machines in the cell (1). Bacterial 70S ribosomes consist of the large 50S and small 30S subunits (1–3). The large 50S subunit, which contains the 23S and 5S rRNAs, binds aminoacyl tRNA, catalyzes peptidyl transfer, and participates in polypeptide elongation. The small 30S subunit, which consists of the 16S rRNA, recognizes mRNA, ensures decoding fidelity, and initiates protein synthesis. The mature 70S ribosome includes the A, P, and E tRNA-binding sites. The A-site allows charged aminoacyl-tRNA to enter the ribosome. The P-site docks peptidyl-tRNA that represents the elongating polypeptide chain. The E-site engages the deacylated tRNA, which is then released from the ribosome. This vast complex machinery offers several binding sites that are potentially “druggable” (4) and is currently targeted by several different classes of protein synthesis inhibitors. Examples include macrolides and oxazolidinones that arrest the P-site of the large 50S subunit and block peptidyl transferase and initiation. Interestingly, both aminoglycosides and tetracyclines are capable of capturing the 30S ribosomal subunits, preventing the binding of tRNA to the A-site, and inhibiting translation, although their mechanisms of action are quite distinct from each other (4, 5).

The tetracycline class of antibiotics are powerful antimicrobials that exhibit inhibition of Gram-positive, Gram-negative, and anaerobic bacteria (6–8). Tetracyclines inhibit bacterial translation through interactions with the small 30S subunit of the bacterial ribosome and block attachment of tRNA to the acceptor A-site, halting elongation of the peptide chain (9). Eravacycline (Era), a fully synthetic fluorocycline containing the tetracyclic core scaffold with modifications in the tetracyclic D ring, shows enhanced broad-spectrum antibacterial activity in comparison to previous generations of tetracycline-based antibiotics (10–13).

Unfortunately, the potency of these antibiotics will eventually diminish as antimicrobial resistance to these drugs increases over time through their overuse and misuse (14). The most prominent tetracycline resistance methods employed by multidrug-resistant (MDR) bacteria are the utilization of multidrug efflux pumps and the expression of ribosomal protection proteins (8, 9, 15). An example of a bacterial strain that has gained substantial antibiotic resistance over time is Acinetobacter baumannii, a Gram-negative opportunistic pathogen. A. baumannii poses a severe nosocomial threat and is listed by the World Health Organization as a first priority for antibiotic research and development (16–18).

Drug resistance in A. baumannii and other MDR bacteria is commonly acquired through the upregulation of the resistance-nodulation-cell division (RND) superfamily of transporters (19, 20). These powerful multidrug efflux systems typically form large tripartite complexes that span the entire cell envelope, including the inner membrane, periplasm, and outer membrane of Gram-negative bacteria (21), allowing for the extrusion of antibiotics and other substrates directly from the cell. In A. baumannii, the tripartite Acinetobacter drug efflux (Ade)IJK efflux system is the most prominent in conferring tetracycline resistance (22–27). AdeJ, the trimeric inner membrane component of the tripartite system, is responsible for drug recognition and proton motive force (PMF) generation that provides the cellular energy required for substrate transport (28). AdeJ works in conjunction with AdeI, the periplasmic membrane fusion protein, and AdeK, the outer membrane channel, to actively export antimicrobials out of the cell (28).

In order to better understand how resistance is conferred to tetracyclines via the A. baumannii AdeJ multidrug efflux pump, we utilized the technique of cryo-electron microscopy (cryo-EM) to solve the structures of the AdeJ pump in the presence of Era. We also acquired structural information of Era-bound forms of the A. baumannii ribosome to critically examine the drug-binding modes of AdeJ and the ribosome. Here, we present the first structures of the A. baumannii AdeJ multidrug efflux pump with and without Era to resolutions of 2.86 Å and 2.87 Å, respectively. We also report six different structural states of the A. baumannii ribosome, including the 70S, 50S, and 30S forms, in the presence of Era, to resolutions that range from 2.50 Å to 3.04 Å. Our results show that the A. baumannii AdeJ pump and ribosome use very distinct modes of action to bind Era, providing a framework for the development of future generations of tetracycline antibiotics optimized to specifically bind the ribosome to halt protein synthesis yet be refractory to RND transporter export.

## RESULTS

### Cryo-EM structure of the A. baumannii AdeJ multidrug efflux.

**FIG 1:**
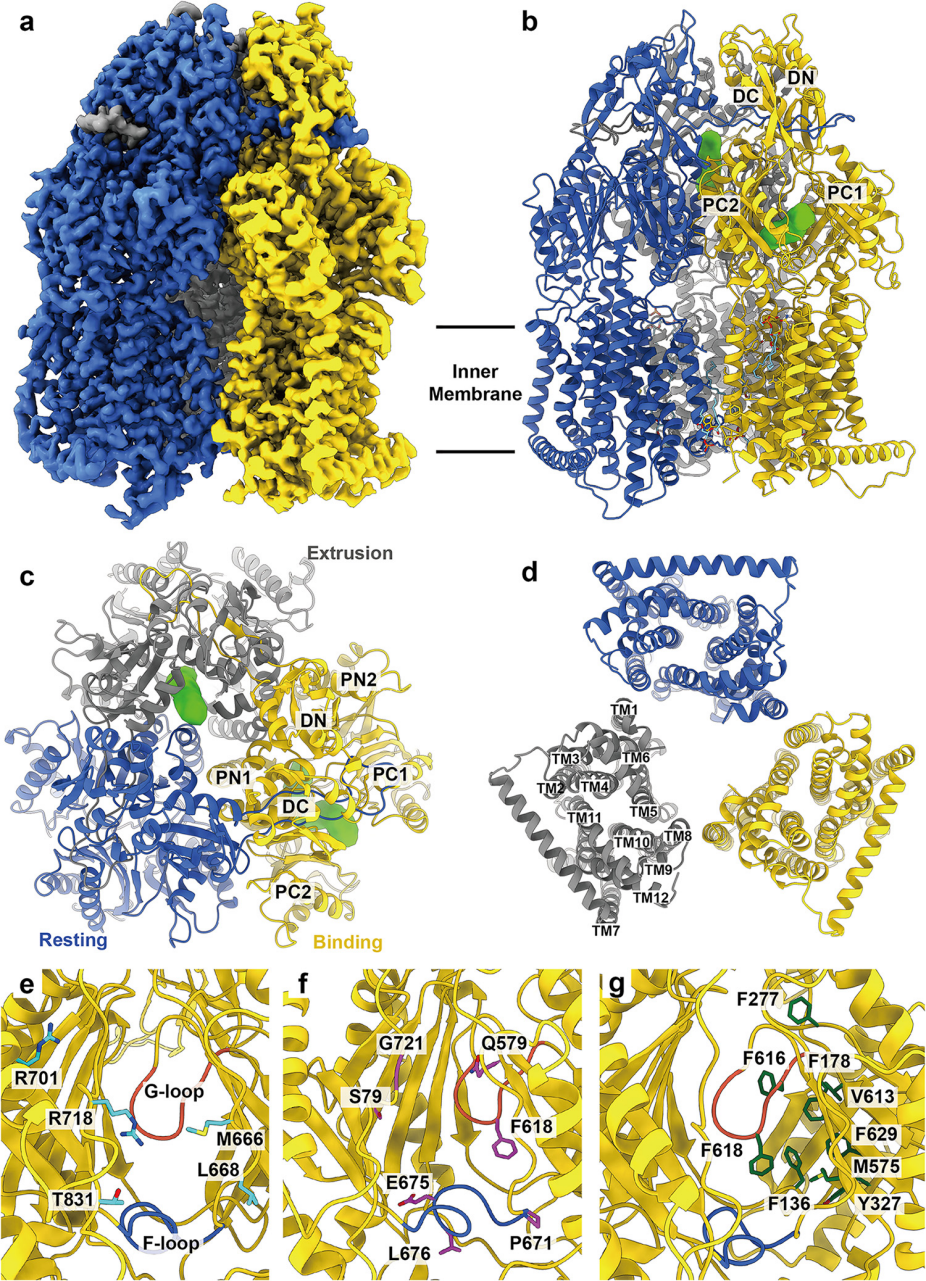
Cryo-EM structure of apo-AdeJ. (a) Side view of the sharpened cryo-EM map of apo-AdeJ (viewed in the membrane plane). (b to d) Ribbon diagram of the structure of the side view (viewed in the membrane plane), top view (viewed from the extracellular space), and bottom view (viewed from the cytoplasm) of the apo-AdeJ trimer. In panels a to d, the resting, binding, and extrusion state protomers are colored royal blue, gold, and gray, respectively. The binding and extrusion channels are colored green. (e) Entrance drug-binding site. (f) Proximal drug-binding site. (g) Distal drug-binding site. In panels e to f, residues that are predicted to be important for selectivity are shown as sticks. The F-loop and G-loop are colored royal blue and tomato, respectively.

**FIG 2:**
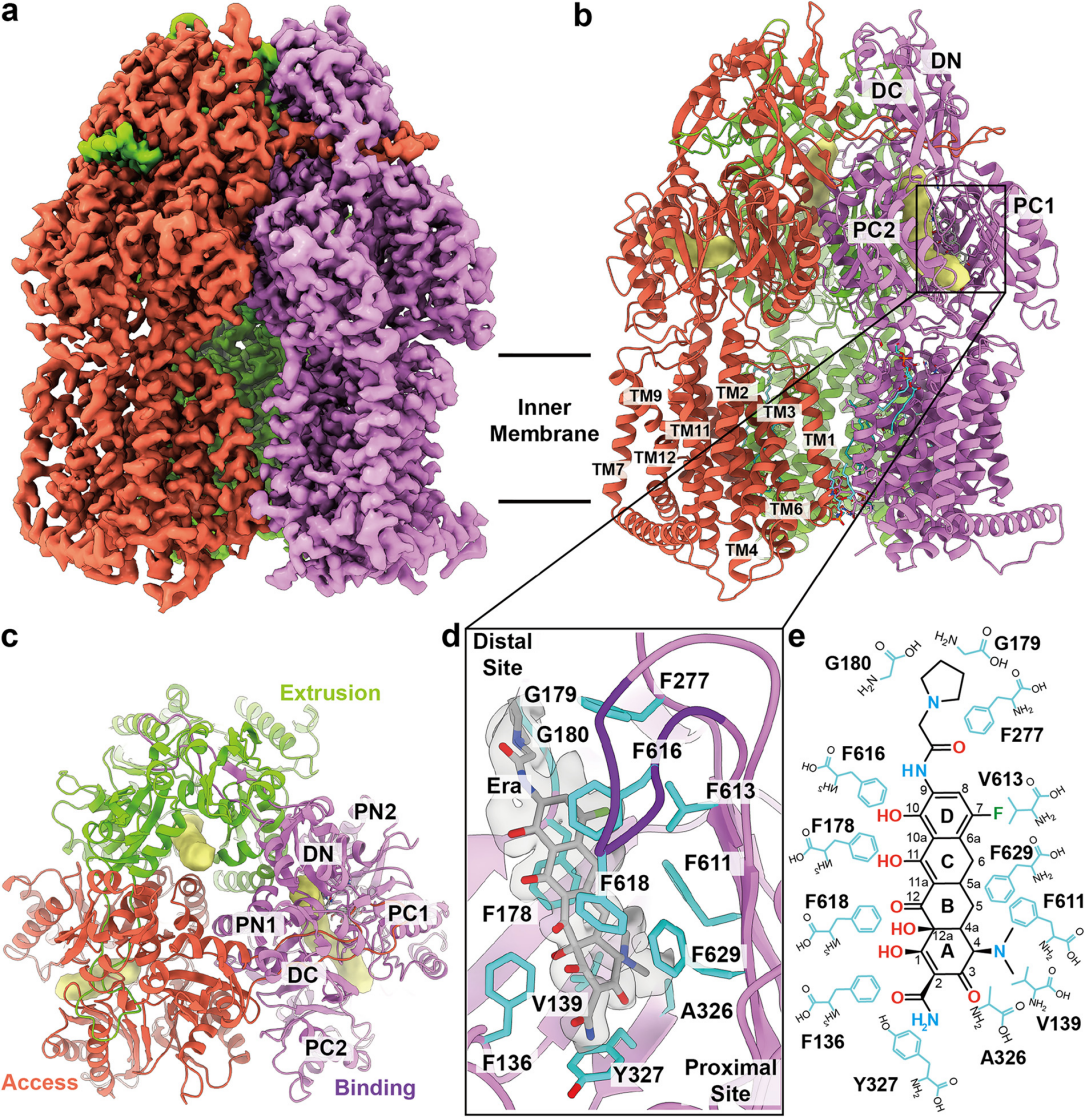
Cryo-EM structure of AdeJ-Era. (a) Side view of the sharpened cryo-EM map of AdeJ-Era (viewed in the membrane plane). (b to c) Ribbon diagram of the structure of the side view (viewed in the membrane plane) and top view (viewed from the extracellular space) of the AdeJ-Era trimer. In panels a to c, the access, binding, and extrusion state protomers are colored in tomato, violet, and lawn green, respectively. The access, binding, and extrusion channels are colored yellow. (d) Era-binding site. Residues that participate in Era binding are shown as cyan sticks. The cryo-EM density of Era is colored gray. The G-loop is colored dark violet. (e) Schematic diagram of the Era-binding site. Amino acids that are important for Era binding are shown as cyan sticks.

To understand how A. baumannii AdeJ recognizes the tetracycline class of antibiotics, we determined its structure using single-particle cryo-electron microscopy (cryo-EM). AdeJ was expressed in Escherichia coli, purified, and reconstituted into lipidic nanodiscs. Subsequently, the AdeJ-nanodisc sample was incubated with Era for 1 h to form the AdeJ-Era complex. Extensive classification of the single-particle images indicated that there were two distinct populations of the AdeJ membrane protein coexisting in this single nanodisc sample (Fig. S1 in the supplemental material). Several iterative rounds of classifications allowed us to sort the images based on these two distinct classes. One of the structures depicted that the trimeric AdeJ pump does not contain any ligands. For the other structure, it was observed that one of the AdeJ protomers within the trimer possesses a bound Era molecule. We then solved the first structures of apo-AdeJ (Fig. 1a to d) and AdeJ-Era (Fig. 2a to c). Of the 1,058 residues of the full-length AdeJ membrane protein, 1,046 residues were built into each protomer.

10.1128/mBio.01031-21.1 FIG S1 Data processing of A. baumannii AdeJ. (a) Data processing workflow of apo-AdeJ and AdeJ-Era. (b and c). Representative two-dimensional classes of apo-AdeJ and AdeJ-Era. (d and e). Gold-standard Fourier shell correlation (GS-FSC) curves of apo-AdeJ and AdeJ-Era. (f and g). Side and top views of the apo-AdeJ and AdeJ-Era cryo-EM density maps. The three protomers in apo-AdeJ are colored royal blue, gold, and gray. The three protomers in AdeJ-Era are colored tomato, violet, and lawn green. Download FIG S1, JPG file, 1.5 MB. Copyright © 2021 Zhang et al. 2021 Zhang et al. https://creativecommons.org/licenses/by/4.0/ This content is distributed under the terms of the Creative Commons Attribution 4.0 International license.

Overall, AdeJ adopts the fold of a typical hydrophobe-amphiphile efflux (HAE)-RND efflux pump and assembles as a homotrimer (Fig. 1b to d). Each protomer can be divided into periplasmic and transmembrane regions. The periplasmic region contains six subdomains, PN1, PN2, PC1, PC2, DN, and DC. Subdomains PN1, PN2, PC1, and PC2 form the portal domain, whereas DN and DC form the docking domain. Consistent with the structures of other RND-inner membrane pumps, the transmembrane region of AdeJ consists of 12 transmembrane helices (TM1 to TM12).

### Structure of apo-AdeJ.

Our cryo-EM structure of the apo form of AdeJ has provided a clear view of the structural basis of this pump that enables substrate transport (Fig. 1a to d and Table S1). A cleft that forms an entrance drug-binding site is created between subdomains PC1 and PC2, where this cleft can be open or closed. Presumably, a molecule recognized by the entrance drug-binding site will be guided by the flexible loop (F-loop) to travel to the proximal drug-binding site. The substrate will then pass through the gate loop (G-loop) and be delivered to the distal drug-binding site for extrusion (Fig. 1e to g).

10.1128/mBio.01031-21.6 TABLE S1 AdeJ cryo-EM data collection and refinement statistics. Download Table S1, PDF file, 0.03 MB. Copyright © 2021 Zhang et al. 2021 Zhang et al. https://creativecommons.org/licenses/by/4.0/ This content is distributed under the terms of the Creative Commons Attribution 4.0 International license.

The entrance of the AdeJ periplasmic cleft is surrounded by residues M666, L668, R701, R718, and T831 (Fig. 1e). These residues likely play a role in substrate specificity and selectivity. R718 of AdeJ corresponds to R716 and R717 in the periplasmic cleft entrance of Pseudomonas aeruginosa MexB and E. coli AcrB, respectively. These arginines have previously been shown to be important for substrate specificity (29, 30).

There is a conserved F-loop (residues 671 to 680) connecting the cleft entrance to the proximal drug-binding site. This F-loop also creates the bottom section of the proximal site. Cryo-EM structures and molecular dynamics (MD) simulations of the AdeB pump depict this loop as being very flexible with distinct conformations within different physiological states of the pump (31). Interestingly, the corresponding F-loop residues in the CusA heavy metal efflux pump forms a horizontal helix that is critical for Cu(I) and Ag(I) recognition (32–34, 69).

As seen from the E. coli AcrB multidrug efflux pump, there are at least 22 residues that make up the proximal drug-binding site of AcrB (35). It is expected that the corresponding residues within AdeJ are responsible for generating its proximal drug-binding site. Interestingly, these residues, including S79, Q579, F618, E675, L676, G671, and G721, are conserved among A. baumannii AdeJ, A. baumannii AdeB (31, 36), E. coli AcrB (37, 38), and Neisseria gonorrhoeae MtrD (39, 40). In addition, the two other AdeJ residues, M575 and R718, are conserved among the AdeJ, AcrB, and MtrD pumps.

The conserved G-loop (residues 615 to 624) compartmentalizes the proximal and distal drug-binding sites. Similar to the F-loop, the cryo-EM structures and MD simulations of the AdeB pump indicate that this G-loop is highly flexible (31). This loop likely participates in the transfer of drug molecules from the proximal to the distal site. Notably, the conserved phenylalanine, F612, of the G-loop of AdeB is involved in drug binding at the distal site (31).

In the E. coli AcrB pump, the distal drug-binding site has been reported to contain at least 23 residues (35). Of these 23 residues, 6 are conserved among AdeJ, AdeB, AcrB, and MtrD (31, 36–40). These AdeJ residues correspond to F136, F178, Y327, V613, F618, and F629. In addition, several AdeJ residues important for drug recognition are partially conserved. The AdeJ residue F277 is conserved with AdeB (31, 36) and AcrB (37, 38), whereas the AdeJ residues M575 and F616 are conserved with AcrB (37, 38) and MtrD (39, 40). Four of these residues, F178, F277, V613, and F616, form a hydrophobic patch within the distal site of the AdeJ pump. It is expected that this hydrophobic patch plays a critical role in drug binding and export, as seen in the corresponding patch of the AcrB efflux pump.

In the apo-AdeJ structure, the periplasmic cleft of one of the protomers is open. However, the clefts of the other two protomers are closed. A horizontal “binding” channel, mostly in parallel with the membrane surface, is found in the periplasmic domain of the protomer with the cleft open. This channel leads through the open cleft, allowing the interior of the periplasmic domain exposure to solvent. This conformer represents the binding state of AdeJ (Fig. 1c). The assignments of the AdeJ protomers are shown in Fig. S2 and Table S2.

10.1128/mBio.01031-21.2 FIG S2 AdeJ structural state assignment. (a). Distance between DN and DC domains (exit tunnel) in AdeJ-Era (top). The access, binding, and extrusion state protomers are colored violet, tomato, and lawn green, respectively. DN and DC domain distance of the three apo-AdeJ protomers (bottom). The resting, binding, and extrusion state protomers are labeled in royal blue, gold, and gray, respectively. Distances are measured between the Cα atoms of Q125 and Y759. (b) Proton relay network of AdeJ. Resting, access, binding, and extrusion states of AdeJ protomers are colored royal blue, tomato, violet, and lawn green, respectively. Residues involved in the proton relay network (D407, D408, K952, N953, and T989) are showed as cyan sticks and surface density. Hydrogen bonds between K952 and D407, D408, K953, and T989 are labeled with yellow dashes. Download FIG S2, JPG file, 1.4 MB. Copyright © 2021 Zhang et al. 2021 Zhang et al. https://creativecommons.org/licenses/by/4.0/ This content is distributed under the terms of the Creative Commons Attribution 4.0 International license.

10.1128/mBio.01031-21.7 TABLE S2 Classification of AdeJ protomer states. Download Table S2, PDF file, 0.03 MB. Copyright © 2021 Zhang et al. 2021 Zhang et al. https://creativecommons.org/licenses/by/4.0/ This content is distributed under the terms of the Creative Commons Attribution 4.0 International license.

For one of the protomers with the periplasmic cleft closed, a vertical “extrusion” channel perpendicular to the membrane surface is observed in the periplasmic domain. This conformer presents the extrusion state of the pump (Fig. 1c). No channel can be discerned in the third protomer, which also has its periplasmic cleft closed. This conformer depicts the “resting” state of the pump (Fig. 1c). Interestingly, the conformational states of the three AdeJ protomers within the trimer resemble the crystal structure of the Campylobacter jejuni CmeB trimeric multidrug efflux pump (41), where the three protomers represent the resting, extrusion, and binding conformations of this membrane protein.

### Structure of AdeJ-Era.

Interestingly, the cryo-EM structure of AdeJ-Era indicates that this pump-antibiotic complex forms an asymmetric trimer where the three protomers are distinct and display different conformational states (Fig. 2a to c, Fig. S1, and Table S1). The AdeJ-Era trimer structure is also distinct from that of trimeric apo-AdeJ. Superimposition of the AdeJ-Era and apo-AdeJ trimers results in an overall root mean square deviation (r.m.s.d.) of 1.2 Å (Fig. S3). Notably, the periplasmic clefts of two AdeJ protomers are open, whereas the cleft of the third protomer remains closed. Like the structures of typical asymmetric pumps found in E. coli AcrB (42) and N. gonorrhoeae MtrD (40), the conformations of these three AdeJ protomers can be classified as the access, binding, and extrusion forms, respectively. The corresponding access, binding, and extrusion channels are seen in these protomers (Fig. 2c).

10.1128/mBio.01031-21.3 FIG S3 Superimposition of the structures of apo-AdeJ and AdeJ-Era. (a) The r.m.s.d. between the apo-AdeJ and AdeJ-Era is 1.206 Å. The resting, binding, and extrusion protomers of apo-AdeJ are colored blue, gold, and gray, respectively. The access, binding, and extrusion protomers of AdeJ-Era in tomato, violet, and lawn green, respectively. The helices are shown as tubes. (b to d) The r.m.s.d. values between the resting protomer of apo-AdeJ and access protomer of AdeJ-Era (b), binding protomer of apo-AdeJ and binding protomer of AdeJ-Era (c), and extrusion protomer of apo-AdeJ and extrusion protomer of AdeJ-Era (d) are shown. (e) Enlarged view of the distal drug-binding site of the apo-AdeJ-binding protomer (gold) and AdeJ-Era-binding protomer (violet). The motion of the G-loop between these two protomers is highlighted with a black arrow. Download FIG S3, JPG file, 1.4 MB. Copyright © 2021 Zhang et al. 2021 Zhang et al. https://creativecommons.org/licenses/by/4.0/ This content is distributed under the terms of the Creative Commons Attribution 4.0 International license.

In the binding protomer of AdeJ-Era, a large extra density is found deep inside the open periplasmic cleft of this protomer. This large density corresponds to the bound Era drug located at the distal multidrug-binding site of the pump (Fig. 2d). The Era molecule is embedded in a hydrophobic environment where the pump-antibiotic interactions are mostly hydrophobic in nature. The binding includes the involvement of 13 amino acids, specifically F136, V139, F178, G179, G180, F277, A326, Y327, F611, V613, F616, F618, and F629, to anchor the Era molecule (Fig. 2d and e). Detailed analysis shows that F178 and F629 make π-alkyl interactions with the C and A rings of Era, respectively. Additionally, F616 forms a π-π stacking interaction with the D ring of Era, whereas F277 stabilizes the pyrrolidinoacetamido group of Era using a π-alkyl interaction. Further, V139, A326, and F611 form alkyl-alkyl and π-alkyl interactions with the dimethylamine group of Era. The hydroxyl group of Y327 also contributes through an electrostatic interaction with Era to further secure the binding (Fig. 2d and e). While the Mg^2+^ ion is known to play a role in stabilizing the binding of tetracyclines to ribosomes, it is surprising that we do not see additional cryo-EM densities corresponding to Mg^2+^ ions within the vicinity of bound Era in the AdeJ-Era structure. However, this finding indeed agrees with the previous structural study of AcrB bound with the tetracycline derivative minocycline using X-ray crystallography, where no bound Mg^2+^ ion was found (42).

It is known that RND pumps are powered by the proton motive force (PMF) generated from the transmembrane domain to extrude drugs from the periplasmic domain. In the transmembrane domain of AdeJ, residues D407, D408, K952, N953, and T989 are conserved and form the proton relay network (Fig. S2). These residues are responsible for the transfer of protons from the periplasm to the cytoplasm to generate the PMF necessary for expelling drugs from the cell. Our AdeJ structures clearly indicate the rearrangement of hydrogen bonds formed by side chains of these conserved residues to sequentially switch the conformational state of the AdeJ protomer between the resting, access, binding, and extrusion states (Fig. S2).

### Cryo-EM structures of the A. baumannii ribosome.

Since both the A. baumannii AdeJ pump and 70S ribosome are capable of binding tetracyclines, we decided to solve the cryo-EM structure of A. baumannii 70S bound with Era. These structures are expected to shed light on the mechanisms of drug recognition for these two biomolecules.

The A. baumannii 70S ribosome was incubated with Era for 1 h to form the ribosome-Era complex. We then used cryo-EM to solve structures of this complex. Extensive classification allowed us to separate the single-particle data into six distinct particle classes. The first two particle classes represent images from the small 30S subunit and the large 50S subunit (Fig. S4a to e).

10.1128/mBio.01031-21.4 FIG S4 Data workflow of A. baumannii ribosome. (a) We processed 3,217 micrographs to give an initial pool of 404,703 particles. (b) Extensive three-dimensional classification resulted in three different populations, 50S, 30S, and 70S. (c) Classes were combined and used as targets to retrieve particles from the full particle set. (d) Refined 30S cryo-EM density. (e) Refined 50S cryo-EM density. (f) Three-dimensional variability analysis followed by clustering in cryoSPARC was used to separate 70S particle by tRNA population and hpf occupancy. (g) Final reconstructions of P-site tRNA, E-site tRNA, empty and hpf-bound 70S particles, respectively. Download FIG S4, JPG file, 1.5 MB. Copyright © 2021 Zhang et al. 2021 Zhang et al. https://creativecommons.org/licenses/by/4.0/ This content is distributed under the terms of the Creative Commons Attribution 4.0 International license.

Similar to the previous study of the A. baumannii 70S ribosome (43), we observed particles originating from the P-site tRNA, E-site tRNA, and empty 70S ribosomes where the P- or E-site was found to either be occupied by a tRNA molecule or absent at these sites. In our data set, we also noticed a distinct set of the 70S ribosome images populated with the hibernation-promoting factor hpf, giving rise to the fourth structure of the 70S ribosome (Fig. S4f to g).

### Structure of 30S-Era.

**FIG 3:**
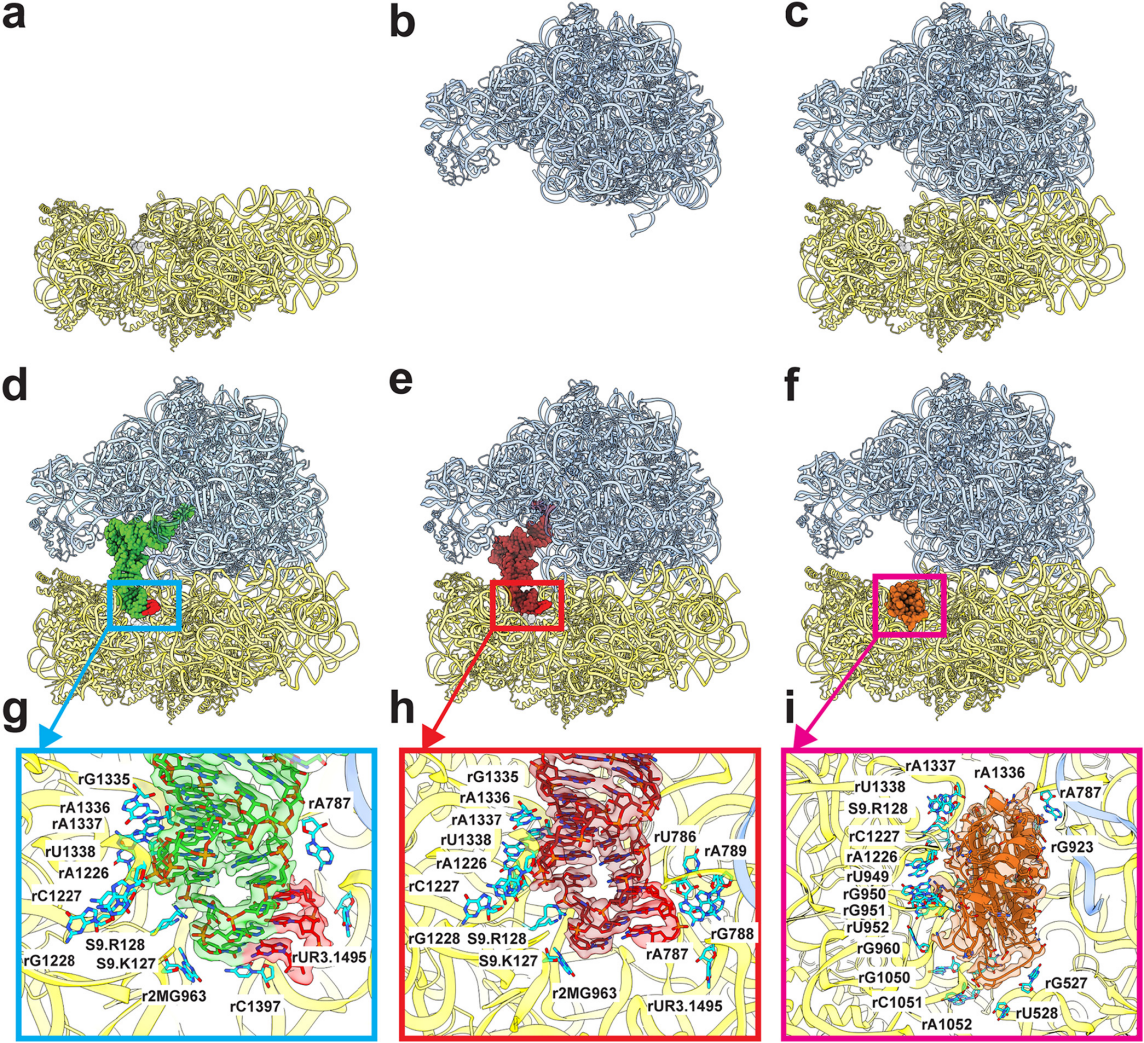
Cryo-EM structures of the A. baumannii ribosome bound with Era. (a to f) Structures of the A. baumannii 30S, 50S, empty 70S, P-site tRNA 70S, E-site tRNA 70S, and hpf-bound 70S. All of these ribosomes are bound with Era. In panels d to f, the P-site tRNA and E-site tRNA are colored green and brown, respectively. The mRNA fragment is in red. The bound hpf is colored orange. (g) P-site tRNA-binding site. The cryo-EM density corresponding to bound tRNA is colored green. The density corresponding to the mRNA fragment is colored red. Nucleotides that are involved in tRNA binding are shown as cyan sticks. (h) E-site tRNA-binding site. The cryo-EM density corresponding to bound tRNA is colored brown. The density corresponding to the mRNA fragment is colored red. Nucleotides that are involved in tRNA binding are shown as cyan sticks. (i) hpf-binding site. The cryo-EM density corresponding to bound hpf is colored orange. Nucleotides that are involved in hpf binding are shown as cyan sticks.

**FIG 4:**
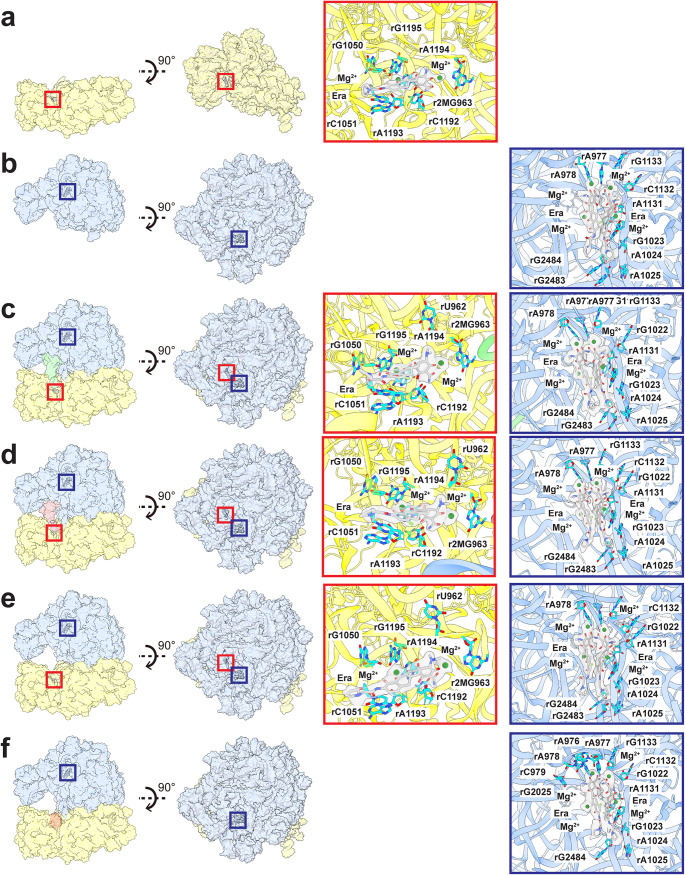
Era-binding sites of the ribosomes. (a) Era-binding site at the 30S subunit. (b) Era-binding site at the 50S subunit. (c) Era-binding sites at the 30S and 50S subunits of the P-site tRNA 70S ribosome. (d) Era-binding sites at the 30S and 50S subunits of the E-site tRNA 70S ribosome. (e) Era-binding sites at the 30S and 50S subunits of the empty 70S ribosome. (f) Era-binding site at the 50S subunit of the hpf-bound 70S ribosome. In panels a to f, the secondary structural elements of the 30S and 50S subunits are colored yellow and blue. The Era-binding site at the 30S subunit is highlighted with a red square. The Era-binding site at the 50S subunit is highlighted with a dark blue square. Nucleotides that are involved in Era binding at either the 30S subunit or 50S subunit are shown as cyan sticks. The cryo-EM densities of the bound Era molecules are colored gray. The bound Mg^2+^ ions are in green spheres.

The structure of the small 30S subunit core was solved to a resolution of 2.80 Å, but the resolution of its head group reached 2.71 Å (Fig. 3a, Fig. S5a, and Table S3). Based on the high-quality cryo-EM maps, we identify the 16S rRNA along with 20 rProteins in this subunit. A large extra density corresponding to bound Era is found nearby the nucleotide rC1051, where this nucleotide, along with r2MG963, rG1050, rC1192, rA1193, rA1194, and rG1195, contribute to stabilize the bound Era via electrostatic interactions (Fig. 4a). In addition, two Mg^2+^ ions are observed to coordinate with the Era molecule. Notably, the Era-binding site overlaps with the previously reported tetracycline-binding site in the 30S subunit (44, 45). This suggests that this specific binding region can accommodate a wide range of tetracycline-based molecules.

10.1128/mBio.01031-21.5 FIG S5 A. baumannii ribosome refinement. (a) Representative two-dimensional classes (top left); GS-FSC curves of two locally refined regions, 30S core and 30S head (right); and combined final density-modified 30S map (bottom left). (b) Representative two-dimensional classes (top left), GS-FSC curve (right), and combined final density-modified 50S map (bottom). (c) Representative two-dimensional classes (top left); GS-FSC curves of three locally refined regions, 50S, 30S core, and 30S head (right); and combined final density-modified P-site tRNA 70S map (bottom). (d) Representative two-dimensional classes (top left); GS-FSC curves of three locally refined regions, 50S, 30S core, and 30S head (right); and combined final density-modified E-site tRNA 70S map (bottom). (e) Representative two-dimensional classes (top left); GS-FSC curves of three locally refined regions, 50S, 30S core, and 30S head (right); and combined final density-modified empty 70S map (bottom). (f) Representative two-dimensional classes (top left); GS-FSC curves of three locally refined regions, 50S, 30S core, and 30S head (right); and combined final density-modified hpf-bound 70S map (bottom). In all panels, the 50S is depicted in blue, 30S core is shown in yellow, and the 30S head is colored red. Download FIG S5, JPG file, 1.8 MB. Copyright © 2021 Zhang et al. 2021 Zhang et al. https://creativecommons.org/licenses/by/4.0/ This content is distributed under the terms of the Creative Commons Attribution 4.0 International license.

10.1128/mBio.01031-21.8 TABLE S3 Ribosome cryo-EM data collection and refinement statistics. Download Table S3, PDF file, 0.04 MB. Copyright © 2021 Zhang et al. 2021 Zhang et al. https://creativecommons.org/licenses/by/4.0/ This content is distributed under the terms of the Creative Commons Attribution 4.0 International license.

### Structure of 50S-Era.

We clearly observe that the large 50S subunit consists of the 23S rRNA, 5S rRNA, and 28 rProteins based on the cryo-EM map (Fig. 3b; Fig. S5b). Surprisingly, two Era molecules bind simultaneously in this 50S subunit (Fig. 4b). Each Era is coordinated with two Mg^2+^ ions. These two Era molecules contact one another to form a dimer via aromatic stacking (Fig. 4b). At least 10 nucleotides of the 23S rRNA, including rA977, rA978, rG1023, rA1024, rA1025, rA1131, rC1132, G1133, rG2483, and rG2484, participate in stabilization of this Era dimer via electrostatic interactions.

### Structure of 70S-Era.

The cryo-EM densities of the four 70S ribosome structures are of high quality, allowing unambiguous structural determination of the 23S rRNA, 5S rRNA, and 28 rProteins from the large 50S subunit and the 16S rRNA and 20 rProteins from the small 30S subunit (Fig. 3c to f; Fig. S5c to f). A tRNA is found to bind at the P-site or E-site within the 70S ribosome structure. The specific interactions between 70S and tRNA are illustrated in Fig. 3g and h. Surprisingly, two distinct Era-binding sites are identified in each of the structures of the P-site tRNA, E-site tRNA, and empty 70S ribosomes (Fig. 4c to e). The binding modes for Era are similar among these three structures. The first Era-binding site is located in the small 30S subunit of 70S, where r2MG963, rG1050, rC1051, rC1192, rA1193, rA1194, and rG1195 contribute to stabilizing Era binding. This binding mode is identical to that of the 30S subunit alone. In the large 50S subunit of 70S, two Era molecules that stack against each other were identified. The nucleotides rA977, rA978, rG1023, rA1024, rA1025, rA1131, rC1132, G1133, rG2483, and rG2484 are responsible for the specific interactions with this Era dimer. As expected, this binding mode is the same as that found in the Era-binding site of the 50S subunit alone.

Interestingly, in the hpf-bound 70S structure, Era densities were only seen in the 50S subunit, but not at the 30S Era-binding site (Fig. 4f). This is likely due to fact that the hpf- and Era-binding sites within the small 30S subunit partially overlap (Fig. 3f and i). It appears that the presence of this hpf molecule inhibits the binding of Era to the 30S subunit. The binding mode of the bound Era dimer at the large 50S subunit is identical to those found in the P-site tRNA, E-site tRNA, and empty 70S structures. Previously, it has been reported that hpf promotes dimerization of 70S to form the 100S ribosome and halts the ribosomal translation process for protein production (55, 67, 68). Our structural data suggest that hpf may also help mediate Era resistance, as Era cannot be bound at the small 30S subunit of the 70S ribosome in the presence of this hibernation-promoting factor. Therefore, the hibernation-promoting factor may use dual mechanisms to protect bacterial cells against unfavorable environmental conditions. These two mechanisms are the formation of 100S ribosome dimers to reduce energy depletion and the association with the 30S subunit to block the action of antimicrobials.

## DISCUSSION

The emergence and spread of drug-resistant A. baumannii have significantly challenged our efforts to treat this infection. Emerging carbapenem-resistant A. baumannii is listed in the highest antimicrobial resistance threat category by the CDC, with carbapenem resistance now found in the majority of A. baumannii strains worldwide. There is also an increasing trend of resistance to polymyxins and tetracyclines, making treatment options very limited. This is due, in part, to the effectiveness of the efflux systems inherent to the bacterium.

We have defined the first structures of the A. baumannii AdeJ multidrug efflux pump both in the absence and presence of Era using cryo-EM (Fig. 1 and 2). Era is bound at the distal drug-binding site deep inside the periplasmic cleft of the AdeJ pump. It appears that AdeJ uses aromatic residues, including F136, F178, F277, Y327, F616, F618, and F629, to bind Era. Interestingly, these conserved phenylalanine residues have also been found to be important for drug recognition in other multidrug efflux pumps, including Escherichia coli AcrB (35, 42, 46), Neisseria gonorrhoeae MtrD (40), and A. baumannii AdeB (31). It should be noted that F178, F277, and F616 are involved in forming the hydrophobic patch, which likely plays a significant role in drug recognition. Therefore, aromatic and hydrophobic interactions may be a general mechanism for these pumps to recognize multiple antimicrobials. The confirmation of the importance of these residues for the function of the AdeJ pump must await further mutagenesis studies.

We have also solved six cryo-EM structures of the A. baumannii ribosome in the presence of Era, including the individual 30S and 50S subunits as well as the complete 70S complex (Fig. 3a to f and Fig. 4a to f). Surprisingly, these structures suggest that the A. baumannii ribosome binds Era disparate from what is seen in AdeJ. Despite differences in assembly (30S, 50S, and 70S) and populations (P-site tRNA, E-site tRNA, empty and hpf-bound 70S particles) (Fig. 3 and 4), Era binding remains consistent, with binding governed by electrostatic interactions. There are at least seven nucleotides in the small 30S subunit involved that interact with the hydroxy and oxo groups of Era. Within the large 50S subunit, the Era-binding site accommodates an Era dimer, where the two Era molecules contact each other via aromatic and hydrophobic interactions. This Era dimer is anchored by at least 10 nucleotides within the 50S. Importantly, electrostatic interaction is the major interacting force between the Era dimer and 50S large subunit.

**FIG 5:**
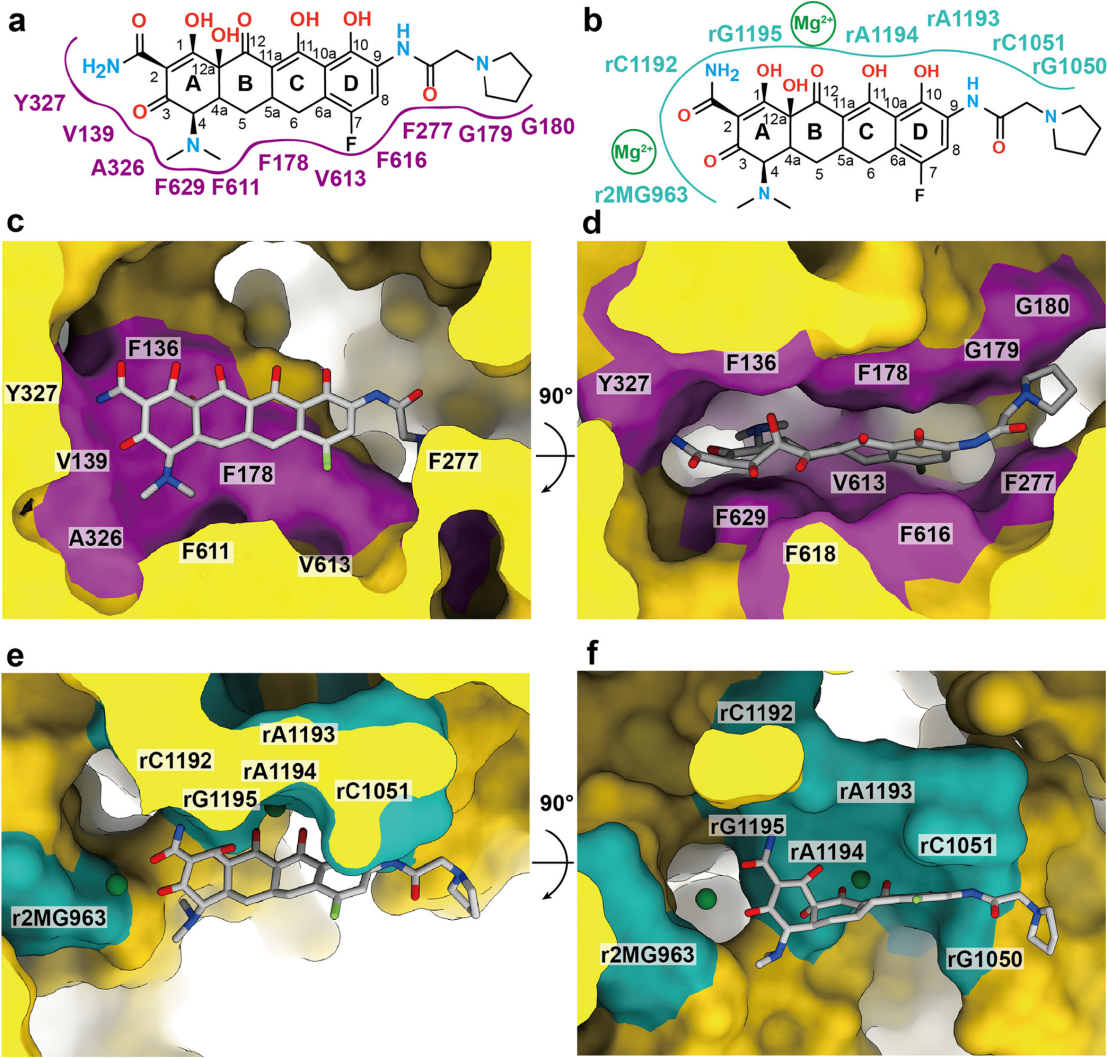
Comparison of the A. baumannii AdeJ and ribosome Era-binding sites. (a) Schematic diagram of the AdeJ Era-binding site. The binding is mainly governed by hydrophobic interactions. (b) Schematic diagram of the 30S subunit Era-binding site. The binding is mainly governed by electrostatic interactions. (c) and (d) AdeJ Era-binding site from the cryo-EM structure. The bound Era molecule is shown as gray sticks. The purple patches represent the locations of residues that are involved in Era binding. (e) and (f) The 30S subunit Era-binding site from the cryo-EM structure. The bound Era molecules are shown as gray sticks. The cyan patches represent the locations of nucleotides that are involved in Era binding. The bound Mg^2+^ ions are shown as green spheres.

In comparison with the AdeJ and 30S Era-binding sites (Fig. 5), it is quite clear that AdeJ utilizes hydrophobic interactions to stabilize Era binding, while the ribosome takes advantage of electrostatic interactions to anchor Era. As the binding modes for Era between AdeJ and ribosome are unique, future drug design can take advantage of their differences by optimizing a tetracycline-based molecule that inhibits bacterial cell function through ribosomal inhibition yet is unable to be recognized and exported by efflux pumps. Our studies may ultimately facilitate structural-guided drug design to combat MDR A. baumannii infections and possibly other MDR bacterial strains as well.

Previous studies indicate that the action mechanism for the tetracycline class of antibiotics is to halt translation elongation via specific interactions with the small subunit 30S of the ribosome. Our structural data agree with this mechanism where Era is observed to specifically interact with 30S, and this Era-binding site also overlaps with the familiar tigecycline-binding site previously found in the A. baumannii 70S ribosome (45). Surprisingly, we also observe a pair of Era molecules which stack against each other to form an Era dimer, bound within the large subunit 50S. The location of this binding site is very different from those found for erythromycin, telithromycin, clindamycin, chloramphenicol, evernimycin, and thiostrepton, where these antibiotics either occupy the peptide exit tunnel, inhabit the tRNA A-site, or hinder the association of the initiation factor IF2 with elongation factors EF-Tu and EF-G (44, 47–53). We observed in the cryo-EM structures that this Era pair occupies a unique binding site in 50S, where it interacts with residues from H40, H89, and the H41/H42 loop of the 23S rRNA. As H89 directly associates with the elbow of the A-site tRNA (54), it is possible that the bound Era pair is able to affect the flexibility of H89 in accommodation of the A-site tRNA. Whether this new binding site is specific for the A. baumannii ribosome invites future experimentation.

It has been documented that one of the major functions of the hibernation-promoting factor hpf is to suppress protein synthesis by promoting the dimerization of ribosomes into the 100S hibernation complex (55, 67, 68). This process is vital for bacterial cell survival, as it helps reduce energy consumption under unfavorable environmental conditions. Surprisingly, we did not observe any single-particle images of the 100S complex in our cryo-EM data. Instead, a class of single-particle images suggests that hpf specifically binds the 70S ribosome, where the hpf-binding site overlaps with the Era-binding site at the 30S subunit. The hpf-bound 70S structure depicts that hpf is capable of blocking the interaction of Era with 30S to halt translation elongation. This suggests that this hibernation-promoting factor can also protect bacterial ribosomes from the antibacterial action of tetracyclines by blocking the binding of this class of drug to the 30S subunit. Our cryo-EM structures suggest that targeting hibernation-promoting factor and prohibiting it to bind 70S may be an effective strategy to combat MDR bacterial infections.

## MATERIALS AND METHODS

### Expression and purification of A. baumannii AdeJ.

The *adeJ* gene encoding the AdeJ multidrug efflux pump of A. baumannii Ab0057 was cloned into the pET15b expression vector in frame with a 6×His tag at the N terminus. The resulting pET15bΩ*adeJ* plasmid was confirmed by DNA sequencing. This plasmid was transfected into E. coli BL21(DE3)Δ*acrB* cells, which harbor a deletion in the chromosomal *acrB* gene. Cells were grown in 6 liters of Luria-Bertani (LB) medium with 100 μg/ml ampicillin at 37°C. When the optical density at 600 nm (OD_600_) reached 0.5, the culture was treated with 0.2 mM isopropyl-β-d-thiogalactopyranoside (IPTG) to induce *adeJ* expression. Cells were then harvested within 4 h of induction. The collected bacteria were resuspended in low-salt buffer (100 mM sodium phosphate, pH 7.4, 10% glycerol, 5 mM ethylenediaminetetraacetic acid [EDTA], and 1 mM phenylmethanesulfonyl fluoride [PMSF]) and then disrupted with a French pressure cell. The membrane fraction was collected and washed twice with high-salt buffer (20 mM sodium phosphate, pH 7.4, 2 M KCl, 10% glycerol, 5 mM EDTA, and 1 mM PMSF) and once with final buffer (20 mM Na-HEPES, pH 7.5, and 1 mM PMSF). The membrane protein was then solubilized in 2% (wt/vol) *n*-dodecyl-β-d-maltoside (DDM). Insoluble material was removed by ultracentrifugation at 100,000 × *g*. The extracted protein was then purified with a nickel-nitrilotriacetic acid (Ni-NTA) column. The purity of the AdeJ protein (>95%) was judged using SDS-PAGE stained with Coomassie brilliant blue. The purified protein was dialyzed against 20 mM Na-HEPES (pH 7.5) and concentrated to 7 mg/ml (60 μM) in a buffer containing 20 mM Na-HEPES (pH 7.5) and 0.05% DDM.

### AdeJ-nanodisc preparation.

To assemble AdeJ into nanodiscs, a mixture containing 20 μM AdeJ, 45 μM MSP (1E3D1), and 930 μM E. coli total extract lipid was incubated for 15 min at room temperature. Afterward, 0.8 mg/ml prewashed Bio-Beads (Bio-Rad) was added. The resultant mixture was incubated for 1 h on ice followed by overnight incubation at 4°C. The protein-nanodisc solution was filtered through a 0.22-μm nitrocellulose filter to remove the Bio-Beads. To separate free nanodiscs from AdeJ-loaded nanodiscs, the filtered protein-nanodisc solution was purified by passage through a Superose 6 column (GE Healthcare) equilibrated with 20 mM Tris-HCl (pH 7.5) and 100 mM NaCl. Fractions corresponding to the size of the trimeric AdeJ-nanodisc complex were collected for cryo-EM.

### Purification of A. baumannii ribosomes.

A. baumannii ribosomes were purified as previously described (43). In brief, A. baumannii (strain AB0057) cells were lysed using a French pressure cell in lysis buffer (20 mM Tris, pH 7.5, 50 mM magnesium acetate [MgOAc], 100 mM NH_4_Cl, 1 mM dithiothreitol (DTT), and 0.5 mM EDTA) (55) and centrifuged at 30,000 × *g* for 30 min. Ribosome-containing pellets were obtained using a sucrose cushion buffer (lysis buffer with 1.1 M sucrose) and centrifuging for 16 h at 100,000 × *g*. The resulting pellet was stored at −80°C until further use.

To increase sample purity, sucrose gradient purification was performed using a sucrose gradient (20 mM HEPES, 14 mM MgOAc, 100 mM KCl, 0.2 mM DTT, and 0.1 mM PMSF with 10% and 40% sucrose and mixed using a gradient maker) and centrifugation for 16 h at 100,000 × *g*. The sample was fractionated and monitored using a plate reader. Fractions containing 70S particles were exchanged into ribosome buffer 2 using 100-kDa cutoff Amicon centrifugal filters (20 mM HEPES-KOH, 10 mM MgOAc, and 100 mM KCl), frozen in liquid nitrogen, and stored at −80°C until further use.

### Electron microscopy sample preparation.

For the AdeJ-Era sample, a 10-μM AdeJ-nanodisc sample was incubated with 20 μM Era (eravacycline dihydrochloride; MedChemExpress) for 1 h to form the AdeJ-Era complex. The sample was applied to glow-discharged holey carbon grids (Quantifoil Cu R1.2/1.3, 300 mesh), blotted for 5 s, and then plunge-frozen in liquid ethane using a Vitrobot (Thermo Fisher). The grids were then transferred into cartridges. The ribosome sample was prepared by incubation of 100 nM A. baumannii ribosome with 50 μM Era for 1 h prior to plunge freezing (15 s blot) on graphene oxide-coated Quantifoil R1.2/1.3 grids. The grids were then transferred into cartridges prior to data collection.

### Data collection.

Cryo-EM data were recorded at a defocus range of −1 to −2.5 μm on a Titan Krios equipped with a K3 direct electron detector (Gatan). For AdeJ, the data were collected in superresolution mode at nominal magnification of 81 K, corresponding to a sampling interval of 1.08 Å/pixel (superresolution, 0.54 Å/pixel). Each micrograph was collected over 40 frames and exposed for 2 s with a total specimen dose of 36 e^−^/Å^2^ using SerialEM (56). For the ribosome sample, data were collected in superresolution mode at nominal magnification of 105 K, corresponding to a sampling interval of 0.848 Å/pixel (superresolution, 0.424 Å/pixel). Each micrograph was collected over 46 frames with an exposure time of 1.2 s and a total dose of 46 e^−^/Å^2^ using SerialEM (56).

### Data processing.

For the AdeJ pump, the superresolution image stacks were aligned and binned by 2 using patch motion followed by contrast transfer function (CTF) estimation by patchCTF in cryoSPARC (57). Initially, 2,665,397 particles were selected after template picking in cryoSPARC. After a reference-free *ab initio* three-dimensional reconstruction, several iterative rounds of heterogeneous refinement were carried out to remove false picks and classes with unclear features, including ice and carbon contamination. The resulting 649,335 particles were then used for three-dimensional classification. Several rounds of nonuniform refinement were performed, where 83,768 and 95,451 particles were classified as the apo-AdeJ pump and AdeJ-Era complex, respectively. Nonuniform refinement followed by local focused refinement using cryoSPARC resulted in 2.87 Å and 2.86 Å global resolution maps for apo-AdeJ and AdeJ-Era based on the gold-standard Fourier shell correlation (FSC), respectively (Table S1 in the supplemental material; Fig. S1). The final cryo-EM density maps were modified by the Resolve Cryo-EM program in PHENIX (58).

For the A. baumannii ribosome, the superresolution image stacks were aligned and binned by 2 using patch motion, and CTF was estimated using patch CTF in cryoSPARC (57). A subset of micrographs was selected using blob picker. These micrographs were used to generate two-dimensional templates for template picker in cryoSPARC, resulting in an initial pool of 813,709 particles. These particles were cleaned using two rounds of two-dimensional classification and classified using three-dimensional *ab initio* and three-dimensional heterogeneous refinement to create the initial 70S, 50S, and 30S maps. Using a modified “build and retrieve” (BaR) (59) method, these maps were used to retrieve particles from the initial 813,709 stack images followed by two-dimensional and three-dimensional cleaning, resulting in a marked increase in overall particle counts for each structure. The 70S classes were further separated using three-dimensional variability analysis based on the tRNA and hibernation-promoting factor occupancies. The final classification resulted in six distinct ribosome structures, including the 70S P-site tRNA, E-site tRNA, empty and hpf-bound structures, as well as the 50S subunit and 30S subunit structures. Refinements were split into three local refinement classes based on the ribosome dynamics, 50S subunit, 30S core, and 30S head (Table S3; Fig. S6 to S11). The structures were locally refined prior to local and global CTF refinement in cryoSPARC and a final round of local refinement with nonuniform sampling. Maps were then modified using Resolve Cryo-EM in PHENIX (60). While individual locally refined maps were used to build the models, composite maps of each structure were created using vop maximum in Chimera (61).

### Model building and refinement.

Model buildings of the structures of AdeJ (apo-AdeJ and AdeJ-Era) and ribosome (30S, 50S, and 70S, including P-site tRNA, E-site tRNA, empty and hpf-bound) were based on the cryo-EM maps. A homology structure of apo-AdeJ was generated based on the atomic coordinates of AdeB (PDB ID 6OWS) (36) using the FFAS server (62). This initial model was then fitted into the density map using Chimera (61). The A. baumannii ribosome structures (43), previously determined from our lab, were used as starting models for solving structures of the ribosome-Era complexes. For the hibernation-promoting factor, the SWISS-MODEL server was utilized to generate a homology model that was subsequently fitted and refined (63). Mg^2+^ ions were manually placed in densities showing clear octahedral coordination with water using the E-site tRNA 70S structure, and positions were transferred to the remaining structures. Only waters coordinated to the Mg^2+^ ions were manually added in Coot (64). Subsequent model buildings and structural refinements were performed using Coot (64) and phenix.real_space_refine (65) from the PHENIX suite (58), respectively. The final structures were evaluated using MolProbity (66). The statistics associated with data collection, three-dimensional reconstruction, and model refinement are included in Tables S1 and S3.

### Data availability.

Atomic coordinates and EM maps for the apo-AdeJ and AdeJ-Era structures have been deposited in the RCSB protein data bank (PDB) under accession codes 7M4Q and 7M4P and in the electron microscopy data bank (EMDB) under accession codes EMD-23664 and EMD-23663, respectively. Atomic coordinates for A. baumannii 30S, 50S, P-site tRNA 70S, E-site tRNA 70S, empty 70S, and hpf-bound 70S have been deposited to the RCSB PDB under accession codes 7M4U, 7M4V, 7M4X, 7M4Y, 7M4W, and 7M4Z and the EMDB under accession codes EMD-23666, EMD-23667, EMD-23669, EMD-23670, EMD-23668, and EMD-23671, respectively.
