# Supplementary figures and images for: Cryo-EM Determination of Eravacycline-Bound Structures of the Ribosome and the Multidrug Efflux Pump AdeJ of Acinetobacter baumannii
Source: mBio. 2021 May 28;12(3):e01031-21. doi: 10.1128/mBio.01031-21 (PMC8263017; doi:10.1128/mBio.01031-21)

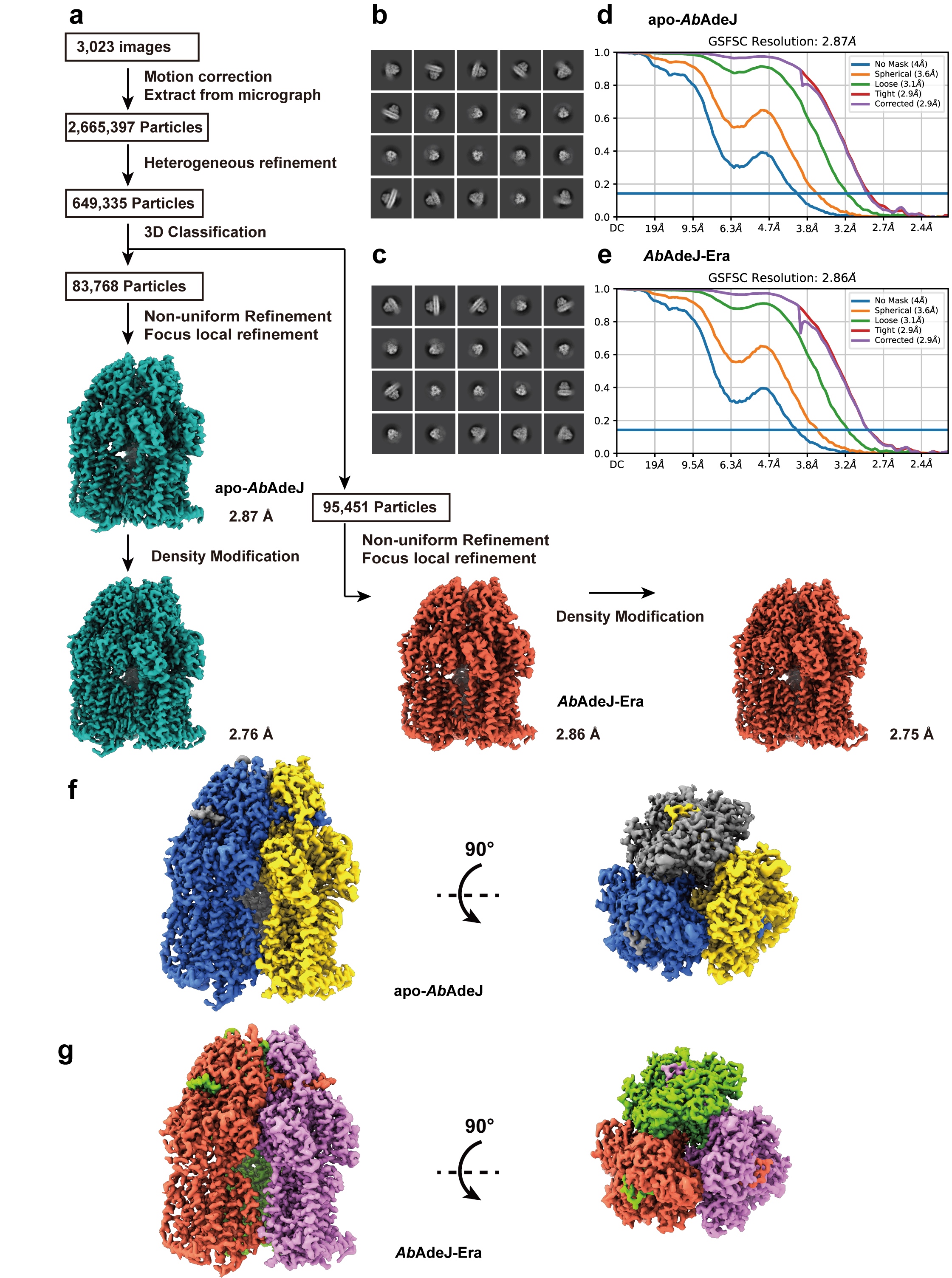

Supplement: FIG S1 [file mbio.01031-21-sf001.jpg]

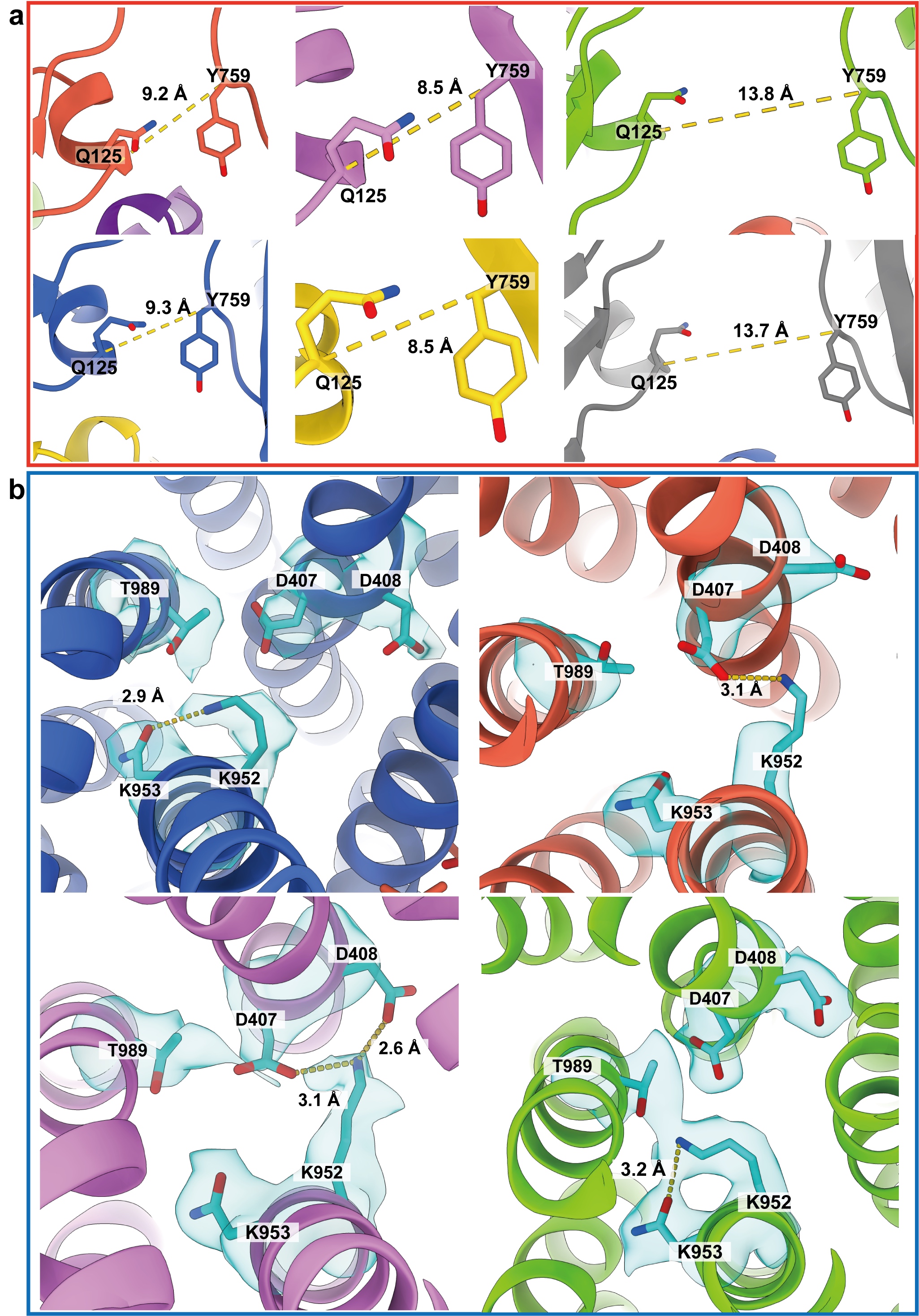

Supplement: FIG S2 [file mbio.01031-21-sf002.jpg]

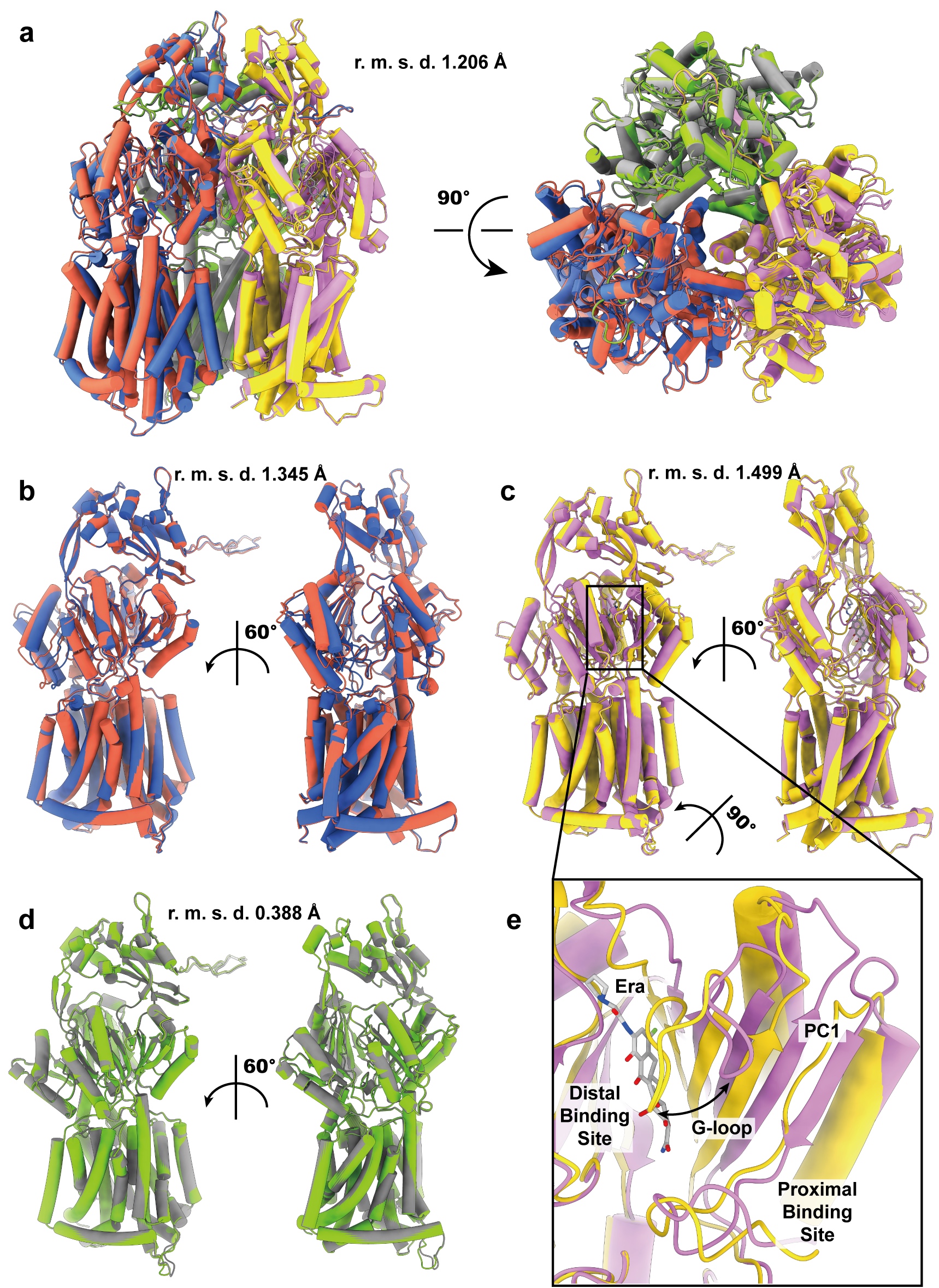

Supplement: FIG S3 [file mbio.01031-21-sf003.jpg]

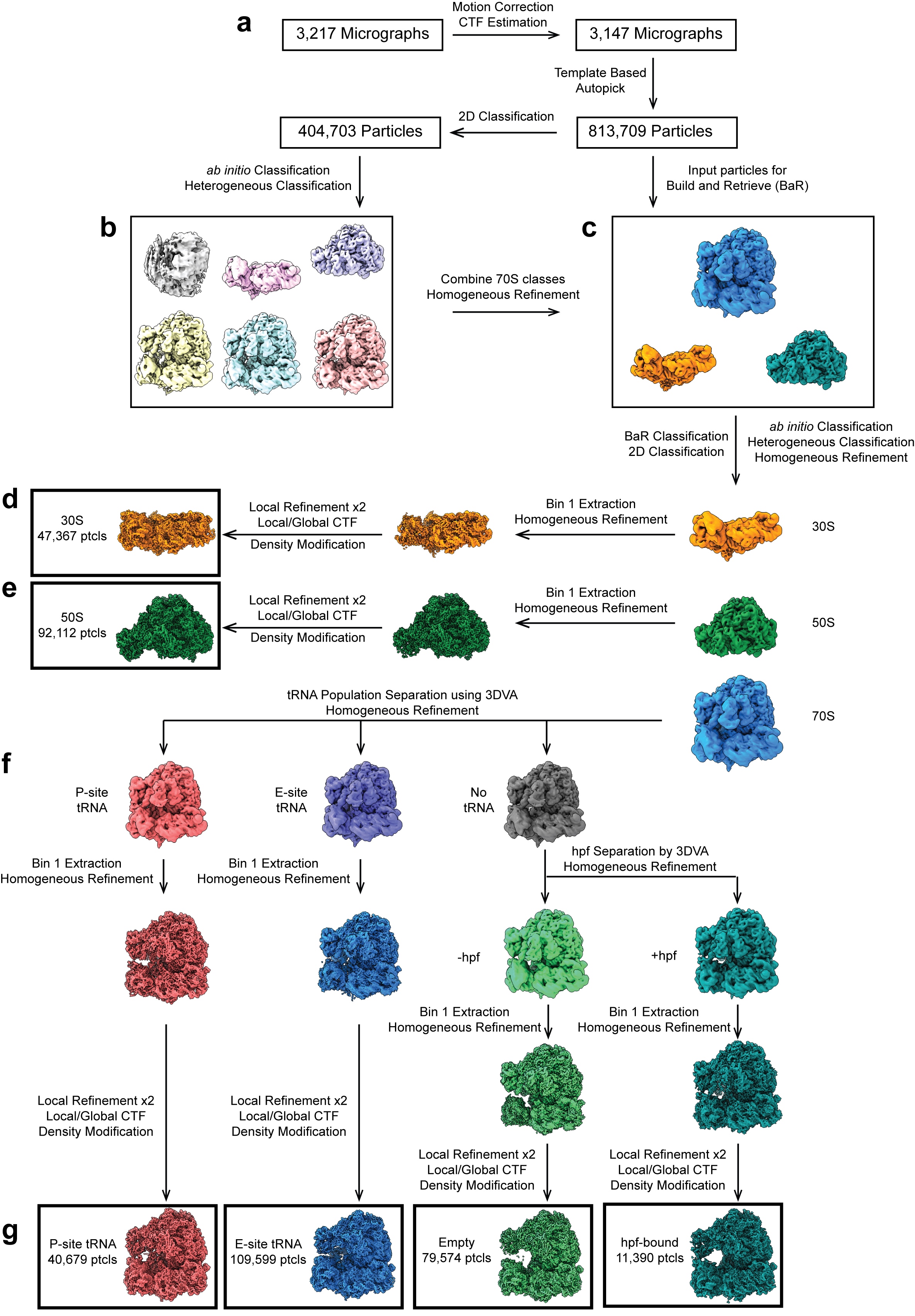

Supplement: FIG S4 [file mbio.01031-21-sf004.jpg]

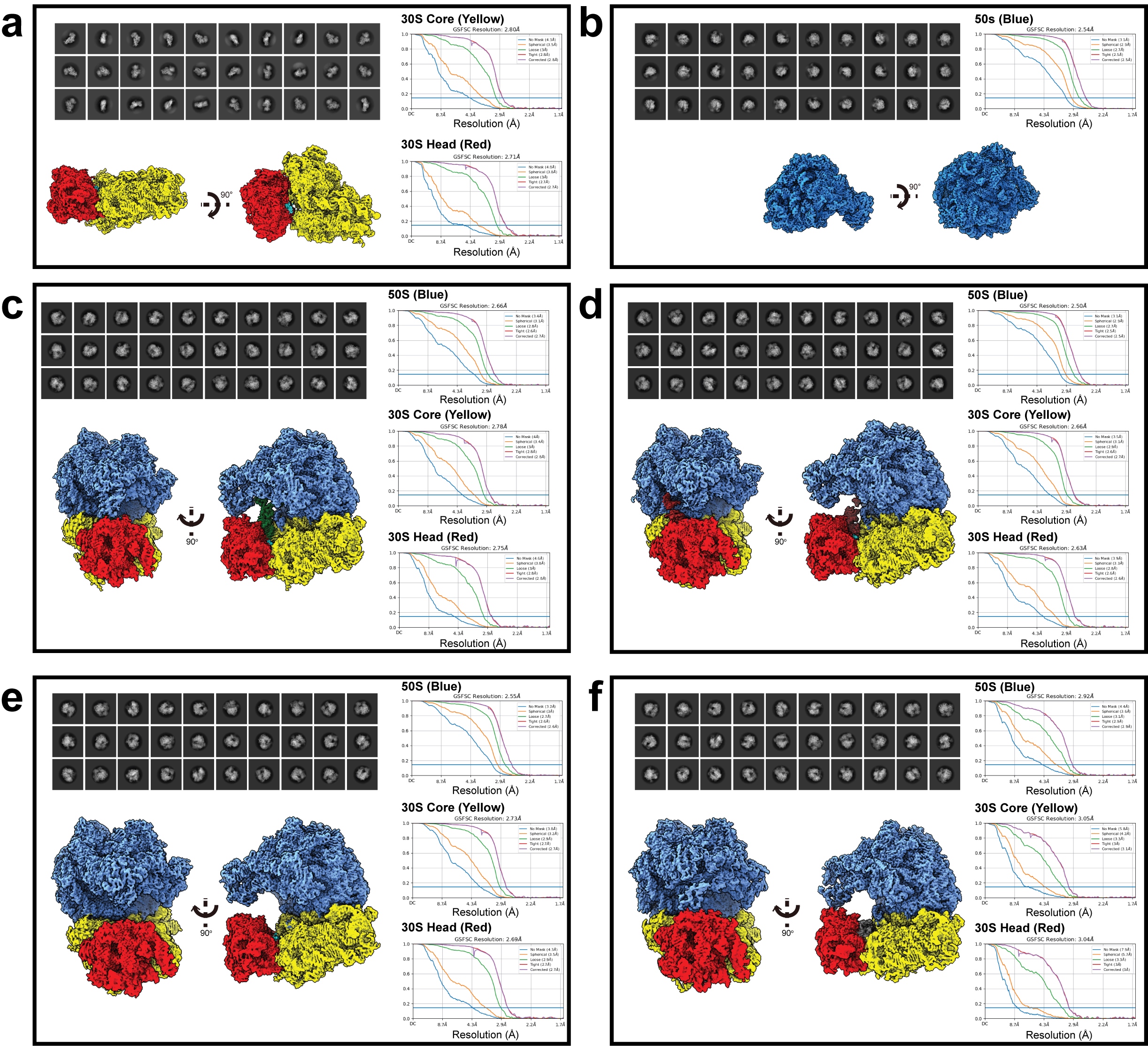

Supplement: FIG S5 [file mbio.01031-21-sf005.jpg]
