# Supplementary material for: Cryo-EM Determination of Eravacycline-Bound Structures of the Ribosome and the Multidrug Efflux Pump AdeJ of Acinetobacter baumannii
Source: mBio. 2021 May 28;12(3):e01031-21. doi: 10.1128/mBio.01031-21 (PMC8263017; doi:10.1128/mBio.01031-21)
Supplement: TABLE S1 [file mbio.01031-21-st001.pdf]

Table S1. AdeJ cryo-EM data collection and refinement statistics.

**Data collection**		
Magnification	81,000	
Voltage (kV)	300	
Electron Microscope	Krios-GIF-K3	
Defocus (um)	-1.0 to -2.5	
Total exposure time (s)	2	
Energy filter width (eV)	20	
Pixel size (Å)	1.08 (0.54)	
Total dose (e^-/ Å^2)	36	
Number of frames	40	
Number of micrographs	3,023	
Initial particle images (no.)	2,665,397	
**Refinement**	**Apo-AdeJ**	**AdeJ-Era**
Total Particles (no.)	83,768	95,451
Symmetry	C1	C1
GS-FSC[a] Resolution (0.143, Å)	2.87	2.86
Model composition		
Protein residues	3,142	3,142
Ligands	8	9
r.m.s.d.		
Bond lengths (Å)	0.010	0.011
Bond angles (°)	0.996	1.074
**Validation**	**Apo-AdeJ**	**AdeJ-Era**
MolProbity score	1.70	1.70
Clash score	5.37	5.82
Ramachandran plot		
Favored (%)	97.54	97.10
Allowed (%)	2.39	2.87
Disallowed (%)	0.06	0.03
CC[b] Mask	0.72	0.72

[a]Gold-Standard Fourier Shell Correlation

[b]Correlation Coefficient
