# Supplementary material for: Cryo-EM Determination of Eravacycline-Bound Structures of the Ribosome and the Multidrug Efflux Pump AdeJ of Acinetobacter baumannii
Source: mBio. 2021 May 28;12(3):e01031-21. doi: 10.1128/mBio.01031-21 (PMC8263017; doi:10.1128/mBio.01031-21)
Supplement: TABLE S2 [file mbio.01031-21-st002.pdf]

Table S2. **Classification of AdeJ protomer states.**

Protomer	Cleft State	Exit site distance, Q125 to Y759	Hydrogen-bonded distance, K952 to				Protomer Assignment
			D407 (Å)	D408 (Å)	N953 (Å)	T989 (Å)	
AdeJ-Apo, A	Closed	9.3		-	2.9	-	Resting
AdeB-Apo, B	Open	8.5	-	2.9	-	-	Binding
AdeB-Apo, C	Closed	13.7	-	-	3.0	3.1	Extrusion
AdeB-Era, A	Open	9.2	3.1	-	-	-	Access
AdeB-Era, B	Open	8.5	3.1	2.6	-	-	Binding
AdeB-Era, C	Closed	13.8	-	-	3.2	-	Extrusion

AdeJ protomers were defined using three criteria; state of the periplasmic cleft (open or closed), measurement of the exit site (distance between Cα atoms of Q125 and Y759) and hydrogen bond distances of the PTC (K952 to D407, D408, N953 and T989). Using these results, protomers were assigned as either resting, access, binding and extrusion.
