# Supplementary material for: Cryo-EM Determination of Eravacycline-Bound Structures of the Ribosome and the Multidrug Efflux Pump AdeJ of Acinetobacter baumannii
Source: mBio. 2021 May 28;12(3):e01031-21. doi: 10.1128/mBio.01031-21 (PMC8263017; doi:10.1128/mBio.01031-21)
Supplement: TABLE S3 [file mbio.01031-21-st003.pdf]

Table S3. Ribosome cryo-EM data collection and refinement statistics.

**Data collection**						
Magnification	105,000					
Voltage (kV)	300					
Electron Microscope	Krios-GIF-K3					
Defocus (um)	-1.0 to -2.5					
Total exposure time (s)	1.2					
Energy filter width (eV)	20					
Pixel size (Å)	0.848 (0.424)					
Total dose (e^-/ $Å^2$)	46					
Number of frames	46					
Number of micrographs	3,217					
Initial particle images (no.)	813,709					
Final particle images (no.)	404,703					
**Refinement**	**30S**	**50S**	**70S P-site-tRNA**	**70S E-site-tRNA**	**70S Empty**	**70S hpf-bound**
Total Particles (no.)	47,367	92,112	40,679	109,599	79,574	11,390
GS-FSC Resolution (0.143, Å)[a]						
50S	-	2.54	2.66	2.50	2.55	2.92
30S Core	2.80	-	2.78	2.66	2.73	3.05
30S Head	2.71	-	2.75	2.63	2.69	3.04
Model composition						
Chains	21	30	53	53	51	52
Protein residues	2,386	3,128	5,528	5,511	5,518	5,625
Nucleotides	1,528	2,847	4,453	4,453	4,373	4,373
Mg^{2+}	83	159	190	196	191	187
Waters	191	802	552	1044	797	229
Era	1	2	3	3	3	2
r.m.s.d.						
Bond lengths (Å)	0.003	0.002	0.002	0.002	0.002	0.002
Bond angles (°)	0.538	0.518	0.501	0.535	0.505	0.507
**Validation**	**30S**	**50S**	**70S P-site-tRNA**	**70S E-site-tRNA**	**70S Empty**	**70S hpf-bound**
MolProbity score	1.43	1.17	1.27	1.51	1.27	1.42
Clash score	4.30	2.88	3.61	3.39	3.05	4.56
Ramachandran plot						
Favored (%)	96.63	97.56	97.37	97.34	96.97	96.85
Allowed (%)	3.15	2.28	2.52	2.49	2.82	2.99
Disallowed (%)	0.21	0.16	0.11	0.17	0.20	0.16
CC Mask	0.75	0.79	0.72	0.72	0.73	0.71

[a]Gold-Standard Fourier Shell Correlation; refinements split into three regions for each structure.
